# Protective Effect of Repeatedly Preadministered Brazilian Propolis Ethanol Extract against Stress-Induced Gastric Mucosal Lesions in Rats

**DOI:** 10.1155/2014/383482

**Published:** 2014-02-03

**Authors:** Tadashi Nakamura, Yoshiji Ohta, Kumiko Ikeno, Koji Ohashi, Takeyuki Ikeno

**Affiliations:** ^1^Japan Beekeeping Co. Ltd., Gifu 500-8691, Japan; ^2^Department of Chemistry, Fujita Health University School of Medicine, Toyoake, Aichi 470-1192, Japan; ^3^Department of Clinical Biochemistry, Faculty of Medical Technology, Fujita Health University School of Health Sciences, Toyoake, Aichi 470-1192, Japan; ^4^Faculty of Health and Nutrition, Shubun University, Ichinomiya, Aichi 491-0938, Japan

## Abstract

The present study was conducted to clarify the protective effect of Brazilian propolis ethanol extract (BPEE) against stress-induced gastric mucosal lesions in rats. The protective effect of BPEE against gastric mucosal lesions in male Wistar rats exposed to water-immersion restraint stress (WIRS) for 6 h was compared between its repeated preadministration (50 mg/kg/day, 7 days) and its single preadministration (50 mg/kg). The repeated BPEE preadministration attenuated WIRS-induced gastric mucosal lesions and gastric mucosal oxidative stress more largely than the single BPEE preadministration. In addition, the repeated BPEE preadministration attenuated neutrophil infiltration in the gastric mucosa of rats exposed to WIRS. The protective effect of the repeated preadministration of BPEE against WIRS-induced gastric mucosal lesions was similar to that of a single preadministration of vitamin E (250 mg/kg) in terms of the extent and manner of protection. From these findings, it is concluded that BPEE preadministered in a repeated manner protects against gastric mucosal lesions in rats exposed to WIRS more effectively than BPEE preadministered in a single manner possibly through its antioxidant and anti-inflammatory actions.

## 1. Introduction

Propolis (bee glue) is a resinous hive product collected by honeybee from various plant sources and it can be used for a wide range of purposes as anti-inflammatory, antioxidant, antibacterial, and immunomodulatory agents [1–3]. Chemically, propolis obtained from different areas of the world is constituted by 50–60% of resin, 30–40% of wax, 5–10% of essential oils, and 5% of pollen, besides microelements [4]. It contains various organic compounds such as phenols, tannins, polysaccharides, terpenes, aromatic acids, and aldehydes [1, 4, 5].

Brazilian green propolis is known to exert antiulcer activity in experimental animal models. It has been reported that the hydroalcoholic extract of Brazilian green propolis, the main phenolic acids in the extract, and *Baccharis dracunculifolia*, the main botanical source of Brazilian green propolis, protect against gastric mucosal lesions induced by ethanol, indomethacin, or water-immersion restraint stress (WIRS) in rats [6–8]. These reports have suggested that the hydroalcoholic extract of Brazilian green propolis could exert an antiulcer effect in rats with WIRS by reducing the volume and acidity of gastric juice and by increasing the pH of gastric juice [6–8].

We and other researches have reported that enhanced lipid peroxidation associated with decreases in the levels of nonprotein sulfhydryl (NPSH), which is mainly reduced glutathione (GSH), vitamin C (VC), that is, ascorbic acid, and vitamin E (VE) and inflammation associated with neutrophil infiltration, excessive reactive oxygen species (ROS) produced via increased xanthine oxidase (XO), and excessive nitric oxide (^•^NO) produced via increased inducible nitric oxide synthase (iNOS) contribute to the development of WIRS-induced gastric mucosal lesions in rats [9–19]. Our previous report has shown that a single oral preadministration of the ethanol extract of Brazilian green propolis protects against gastric mucosal lesions in rats with 6 h of WIRS through its antioxidant properties, although the protective effect of the extract is lower at its dose of 100 mg/kg than at its dose of 50 mg/kg [20]. It has been thought that the higher dose of the ethanol extract of Brazilian green propolis reduces the protective effect against WIRS-induced gastric mucosal lesions by enhancing stress sensitivity [20]. Therefore, it is assumable that repeated oral preadministration of the ethanol extract of Brazilian green propolis to rats with WIRS at a dose of 50 mg/kg/day causes less protective effect against gastric mucosal lesions than a single oral preadministration of the extract at a dose of 50 mg/kg.

In the present study, therefore, we examined whether repeated oral preadministration of the ethanol extract of Brazilian green propolis (50 mg/kg/day) for successive seven days protects against WIRS-induced gastric mucosal lesions in rats in comparison with a single oral preadministration of the extract (50 mg/kg, p.o.). In addition, we evaluated the protective effect of the repeated oral preadministration of the ethanol extract of Brazilian green propolis against WIRS-induced gastric mucosal lesions in rats in comparison with that of a single oral preadministration of VE because it has been reported that a single oral preadministration of VE to rats with WIRS protects against gastric mucosal lesions more effectively than a single oral preadministration of VC through its antioxidant and anti-inflammatory actions [19].

## 2. Materials and Methods

### 2.1. Materials

*RRR*-*α*-tocopherol (*RRR*-*α*-Toc) used for VE administration and 3,3′,5,5′-tetramethylbenzidine were purchased from Sigma (St. Louis, MO, USA) and artepillin C, corticosterone (CORT), *α*,*α*′-dipyridyl, 5,5′-dithiobis(2-nitrobenzoic acid) (DTNB reagent), Folin-Ciocalteu reagent, gallic acid, kaempferol, quercetin, GSH, tocopherol standards such as *α*-tocopherol (*α*-Toc) and *δ*-tocopherol, and other chemicals from Wako Pure Chemical Ind., Ltd. (Osaka, Japan). All chemicals used were of reagent grade and were not further purified.

### 2.2. Preparation of the Ethanol Extract of Brazilian Green Propolis

Brazilian green propolis was collected in the area of Minas Gerais in Brazil by MN Propolis Ind., Comércio e Exportacēo, Ltda (Mogi das Cruzes, SP, Brazil). The quality of the propolis (Lot no. KA-02), which was provided by Japan Beekeeping Co. Ltd. (Gifu, Japan), was as follows: artepillin C, 10.1%; flavonoids, 41.1 mg/g; and bee wax, 5.6%. The ethanol extract of Brazilian green propolis (BPEE) was prepared as described in our previous report [20]. Namely, approximately 35 g of crude propolis was added to 100 mL of 95% ethanol and the mixture was kept at room temperature for 7 days. The final concentration of ethanol and the content of solid components in BPEE were 80% and 13.2%, respectively. This extraction of Brazilian green propolis using 95% ethanol was used in the present study because such ethanol extract of Brazilian green propolis has been reported to exert antioxidant and anti-inflammatory actions in vitro well [21–23] and a protective effect against WIRS-induced gastric mucosal lesions in rats clearly [20].

### 2.3. Chemical Composition Analysis

The content of flavonoid in BPEE was determined by the method of Dowd [24]. The flavonoid content is expressed as that of quercetin equivalents. The content of polyphenol in BPEE was determined by the Folin-Ciocalteu colorimetric method as described by Ahn et al. [25]. The total polyphenol content is expressed as that of gallic acid equivalents. The main constituents in BPEE were analyzed by high-performance liquid chromatography (HPLC) according to the method described by Izuta et al. [21] except that the mobile phase consisting of 1% acetic acid in 55% methanol was replaced by the mobile phase consisting of 1% acetic acid in 69% methanol. The HPLC was performed on a reversed-phase Shim-Pack CLC-ODS (15 cm × 4.5 mm i.d., Shimadzu, Kyoto, Japan) column with water-methanol-acetic acid (30 : 70 : 1, v/v) as a mobile phase at a flow rate of 1 mL/min at 40°C. The volume of the BPEE sample injected to the column was 5 *μ*L. The detection of chlorogenic acid, *p*-coumaric acid, quercetin, cinnamic acid, kaempferol, chrysin, and artepillin C in BPEE was conducted at 290 nm and the content of each constituent was estimated using its authentic compound. The percentages of these constituents in BPEE were calculated from the estimated content of each constituent.

### 2.4. Experimental Animals

Five-week-old male Wistar rats purchased from Nippon SLC Co. (Hamamatsu, Japan) were housed in cages in a ventilated animal room with controlled temperature (23 ± 2°C) and relative humidity (55 ± 5%) with 12 h of light (7:00 to 19:00). The animals were maintained with free access to rat chow, Oriental MF (Oriental Yeast Co., Tokyo, Japan), and tap water ad libitum for one week. All animals received humane care in compliance with the Guidelines of the Management of Laboratory Animals in Fujita Health University. This animal experiment protocol was approved by the Institutional Animal Care and Use Committee and its approved protocol number was M14-02.

### 2.5. Induction and Observation of Gastric Mucosal Lesions

Seven-week-old rats fasted for 24 h were restrained in wire cages and immersed up to the depth of the xiphoid process in a 23°C water bath to induce WIRS-induced gastric mucosal lesions, as described by Takagi and Okabe [26]. Rats were sacrificed under ether anesthesia after 6 h of WIRS. Each isolated stomach filled with 10 mL of 0.9% NaCl was fixed with 10% formalin for 10 min and then cut along the glandular part. The gastric mucosa was carefully examined for lesions recognized as linear breaks (erosions) at the mucosal surface of the glandular part under a stereoscopic microscope (×10). The extent of the lesion (lesion index) is expressed as the sum of the length of these breaks per stomach.

### 2.6. Administrations of BPEE and VE

BPEE was diluted with 5% Tween 80 solution and the diluted BPEE solution contained 6% ethanol. *RRR*-*α*-Toc was dissolved in 5% Tween 80 containing 6% ethanol. Rats aged six weeks were orally preadministered with 1 mL of the diluted BPEE solution, which contained 5 mg of solid components present in the extract, per 100 g body weight every morning (between 9:00 and 9:30), that is, at a dose of 50 mg/kg body weight per day, for successive six days. The rats preadministered repeatedly with BPEE were fasted for 24 h and then exposed to WIRS for 6 h after the final administration of the same dose of BPEE at 30 min before the onset of the stress the following day. All rats without repeated BPEE preadministration were orally administered with 1 mL of 5% Tween 80 containing 6% ethanol (as vehicle) per 100 g body weight once daily at the same time point for successive six days. The vehicle-administered rats with 24 h fasting were orally preadministered with either the diluted BPEE at a dose of 50 mg/kg body weight or *RRR*-*α*-Toc at a dose of 250 mg/kg body weight in the same volume (1 mL/100 g body weight) at 30 min before the onset of WIRS. Unstressed rats without any preadministration (as the control) were orally administered with 1 mL of 5% Tween 80 containing 6% ethanol per 100 g body weight at 30 min before the onset of WIRS. The dose of *RRR*-*α*-Toc used in the present study was determined according to our previous report [19]. The number of rats in the above-described four groups above was 8 each.

### 2.7. Assays of Gastric Mucosal Components and Enzymes and Serum Components

Rats were sacrificed under ether anesthesia at which time blood was collected from the inferior vena cava. Serum was separated from the collected blood by centrifugation. Each stomach isolated after sacrifice was cut along the greater curvature and then the gastric mucosa was collected. The collected gastric mucosa and serum were stored at −80°C until use. Gastric mucosal tissues were homogenized in 9 volumes of ice-cold 50 mM Tris-HCl buffer (pH 7.4) containing 1 mM ethylenediaminetetraacetic acid. The homogenate was used for the assays of NPSH, VE, VC, and lipid peroxide (LPO). NPSH in the homogenate was assayed by the DTNB method of Sedlak and Lindsay [27] using GSH as a standard. VE in the homogenate was assayed by the HPLC method with electrochemical detection using *γ*-tocopherol as an internal standard as described in our previous report [28]. The amount of gastric mucosal VE is expressed as that of *α*-Toc. VC in the homogenate was assayed by the *α*,*α*′-dipyridyl method of Zannoni et al. [29]. LPO in the homogenate was assayed by the thiobarbituric acid method of Ohkawa et al. [30] using tetramethoxypropane as a standard. The amount of gastric mucosal LPO is expressed as that of malondialdehyde (MDA) equivalents. Gastric mucosal NOx (nitrite/nitrate) was assayed by the Griess reaction-dependent method of Green et al. [31]. XO and myeloperoxidase (MPO) in gastric mucosal tissues were assayed by the methods of Hashimoto [32] and Suzuki et al. [33], respectively. For NOx, XO, and MPO assays, the homogenate was sonicated two times on ice for 30 s and then centrifuged at 10,000 ×g for 20 min at 4°C. For NOx assay, the resultant supernatant was further filtrated at 4°C under centrifugation using a membrane filter Ultrafree-MC (Millipore Co., Bedford, MA, USA). NOx in the filtrate was determined using a nitric oxide assay kit (Roche-Diagnostics Co., Tokyo, Japan). XO activity in the supernatant was assessed by measuring the increase in absorbance at 292 nm following the formation of uric acid at 30°C. One unit (U) of this enzyme is defined as the amount of enzyme forming 1 *μ*mol uric acid per min. MPO activity in the supernatant was assessed by measuring the hydrogen peroxide-dependent oxidation of tetramethylbenzidine at 37°C. One unit (U) of this enzyme is defined as the amount of enzyme causing a change in absorbance of 1.0 per min at 655 nm. Serum adrenocorticotropic-stimulating hormone (ACTH) was assayed using a commercial kit, ACTH EIA kit (Phnoix Pharmaceutical Inc., Burlingame, CA, USA). Serum CORT was fluorometrically assayed by the method of Guillemin et al. [34] using authentic CORT as a standard. Serum glucose was assayed using a commercial kit, Glucose C-Test Wako (Wako Pure Chemical Ind., Ltd., Osaka, Japan).

### 2.8. Statistical Analysis

All results obtained are expressed as means ± standard deviation (S.D.). Statistical analyses of the results were performed using a computerized statistical package (StatView). Each mean value was compared by one-way analysis of variance (ANOVA) and Bonferroni/Dunn for multiple comparisons. The significance level was set at *P* < 0.05.

## 3. Results

### 3.1. Chemical Composition in BPPE

The contents of total flavonoid and total polyphenol in BPEE used in the present study were 21.3 and 69.0 mg/g of solid propolis, respectively. The contents of *p*-coumaric acid, kaempferol, chrysin, and artepillin C in the extract were 14.9, 6.75, 2.38, and 47.8 mg/g of solid propolis, respectively. However, no chlorogenic acid, quercetin, and cinnamic acid were detected in the extract. The percentages of *p*-coumaric acid, kaempferol, chrysin, and artepillin C present in BPEE were as follows: *p*-coumaric acid, 0.60%; kaempferol, 0.27%; chrysin, 0.10%; artepillin C, 1.91%.

### 3.2. Gastric Mucosal Lesions

**Figure 1:**
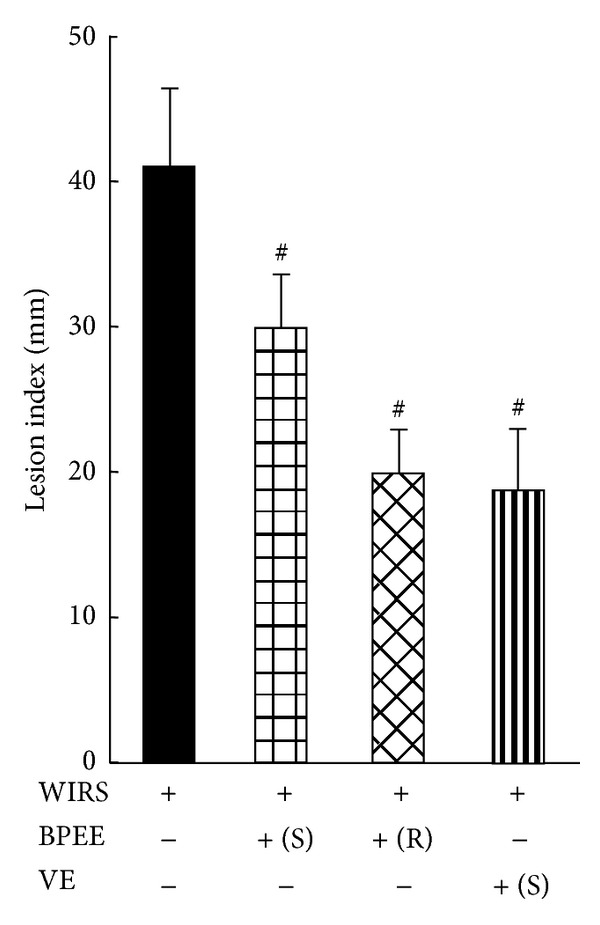
Effects of repeated or a single BPEE preadministration and a single VE preadministration on gastric mucosal lesion development in rats with WIRS. Fasted rats receiving BPEE (50 mg/kg), VE (250 mg/kg) or vehicle at 30 min before the onset of WIRS or BPEE (50 mg/kg) at the same time point after its preadministration for successive six days were exposed to WIRS for 6 h at 23°C as described in Section 2. S and R in the parentheses represent a single manner and a repeated manner of BPEE preadministration, respectively. The lesion index of gastric mucosal tissues was determined as described in Section 2. Each value is a mean ± S.D. (*n* = 8 for each group). ^#^ *P* < 0.05 (versus rats with WIRS alone).

**Figure 2:**
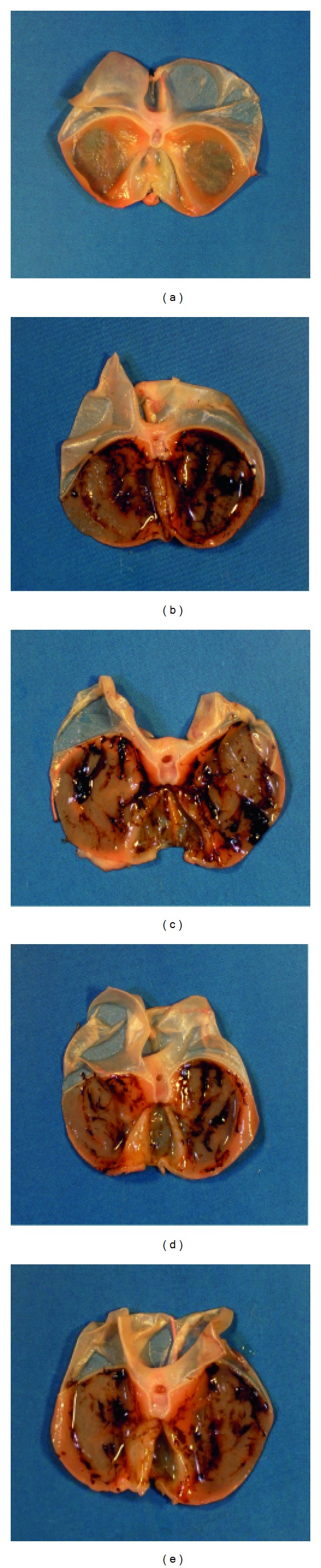
Gross features of typical gastric mucosal lesions in WIRS-exposed rats with and without repeated or a single BPEE preadministration or a single VE preadministration. (a) A control rat without WIRS and any administration; (b) a rat exposed to WIRS alone; (c) a WIRS-exposed rat preadministered once with BPEE (50 mg/kg); (d), a WIRS-exposed rat preadministered repeatedly with BPEE (50 mg/kg/day); (e) a WIRS-exposed rat preadministered once with VE (250 mg/kg).

When rats preadministered with BPEE (50 mg/kg/day, p.o.) for successive seven days or preadministered once with BPEE (50 mg/kg, p.o.) were exposed to 6 h of WIRS, the lesion index in the gastric mucosa of each BPEE-preadministered group was significantly reduced as compared with that of rats with 6 h of WIRS alone (Figure 1). The lesion index in the gastric mucosa of stressed rats with repeated BPEE preadministration was significantly less than that of stressed rats with a single BPEE preadministration (*P* < 0.05) (Figure 1). The lesion index in the gastric mucosa of stressed rats with repeated BPEE preadministration was not significantly different from that of rats exposed to the same period of WIRS after a single preadministration of VE (250 mg/kg, p.o.) (Figure 1). Unstressed control rats orally given 5% Tween 80 containing 6% ethanol as the vehicle showed no gastric mucosal lesion (data not shown).  Figure 2 shows the gross features of typical gastric mucosa lesions in stressed rats without any preadministration, stressed rats preadministered once with BPEE (50 mg/kg), stressed rats preadministered repeatedly with BPEE (50 mg/kg/day, 7 days), and stressed rats preadministered once with VE (250 mg/kg) in comparison with the typical gross feature of the gastric mucosa of unstressed control rats.

### 3.3. Gastric Mucosal NPSH, VC, and VE Concentrations

**Figure 3:**
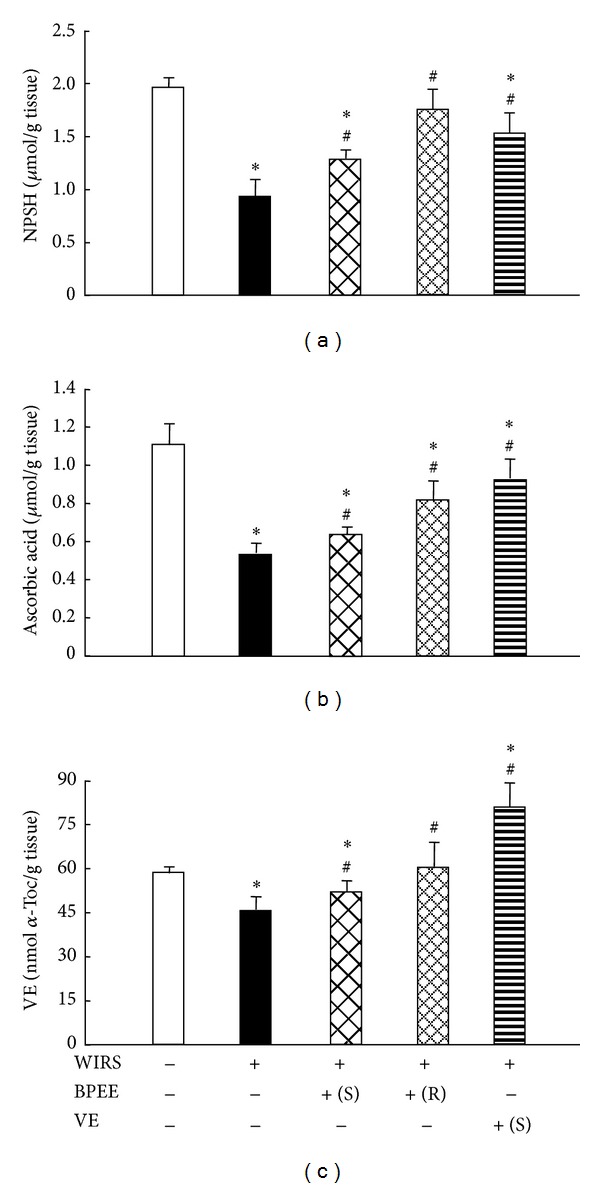
Effects of repeated or a single BPEE preadministration and a single VE preadministration on gastric mucosal NPSH (a), ascorbic acid (b), and VE (c) concentrations in rats with WIRS. Experimental condition and explanation are the same as described in the legend of Figure 1 except that gastric mucosal NPSH, ascorbic acid, and VE were assayed as described in Section 2. Each value is a mean ± S.D. (*n* = 8 for each group). *P < 0.05 (versus control rats without WIRS); ^#^ *P* < 0.05 (versus rats with WIRS alone).

Rats with 6 h of WIRS alone had significantly lower gastric mucosal NPSH, VC, and VE concentrations than unstressed control rats (Figure 3). When rats with 6 h of WIRS were preadministered with BPEE in a single manner or a repeated manner, the decreases in gastric mucosal NPSH, VC, and VE concentrations were significantly attenuated, but the effect of repeated BPEE preadministration to attenuate the decreases in all these gastric mucosal components was significantly larger than that of the single BPEE preadministration (*P* < 0.05) (Figure 3). The gastric mucosal NPSH and VE concentrations in stressed rats with repeated BPEE preadministration were not significantly different from those in unstressed control rats (Figures 3(a) and 3(c)). A single VE preadministration to rats with 6 h of WIRS attenuated the WIRS-induced decreases in gastric mucosal NPSH, VC, and VE concentrations significantly (Figure 3). The gastric mucosal NPSH and VC concentrations in stressed rats preadministered once with VE were almost equal to those in stressed rats preadministered repeatedly with BPEE, although the gastric mucosal VE concentration was significantly higher in the former group than in the latter group (*P* < 0.05) (Figure 3).

### 3.4. Gastric Mucosal LPO and NOx Concentrations and XO and MPO Activities

**Figure 4:**
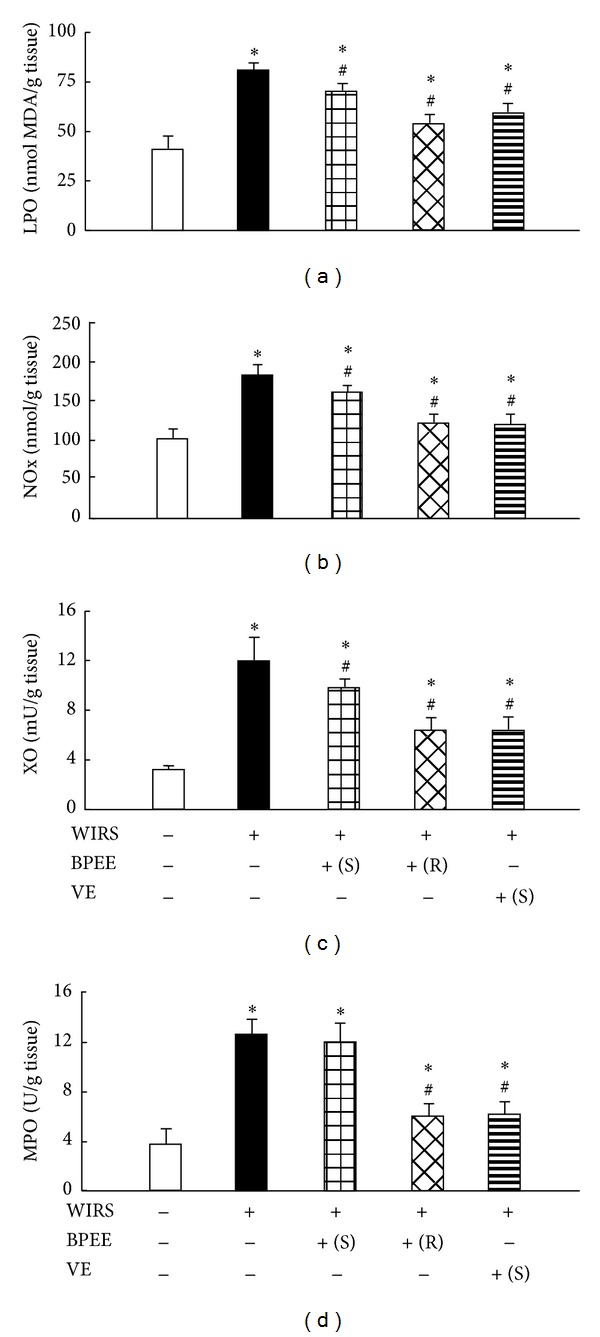
Effects of repeated or a single BPEE preadministration and a single VE preadministration on gastric mucosal LPO (a) and NOx (b) concentrations and XO (c) and MPO (d) activities in rats with WIRS. Experimental condition and explanation are the same as described in the legend of Figure 1 except that gastric mucosal LPO, NOx, XO, and MPO were assayed as described in the Section 2. Each value is a mean ± S.D. (*n* = 8 for each group). *P < 0.05 (versus control rats without WIRS); ^#^ *P* < 0.05 (versus rats with WIRS alone).

Rats with 6 h of WIRS alone showed significant increases in gastric mucosal LPO and NOx concentrations and XO and MPO activities when compared with unstressed control rats (Figure 4). A single or repeated BPEE preadministration to rats with 6 h of WIRS attenuated the increases in gastric mucosal LPO and NOx concentrations and XO activity significantly and the repeated BPEE preadministration further attenuated the increase in gastric mucosal MPO activity significantly (Figure 4). The effect of repeated BPEE preadministration to attenuate the increases in gastric mucosal LPO and NOx concentrations and XO activity was significantly larger than that of a single BPEE preadministration (*P* < 0.05) (Figures 4(a), 4(b), and 4(c)). A single VE preadministration to rats with 6 h of WIRS attenuated the WIRS-induced increases in gastric mucosal LPO and NOx concentrations and XO and MPO activities significantly (Figure 4). There were no significant differences in the gastric mucosal LPO and NOx concentrations and XO and MPO activities between stressed rats with a single VE preadministration and repeated BPEE preadministration (Figure 4).

### 3.5. Serum ACTH, CORT, and Glucose Concentrations

**Figure 5:**
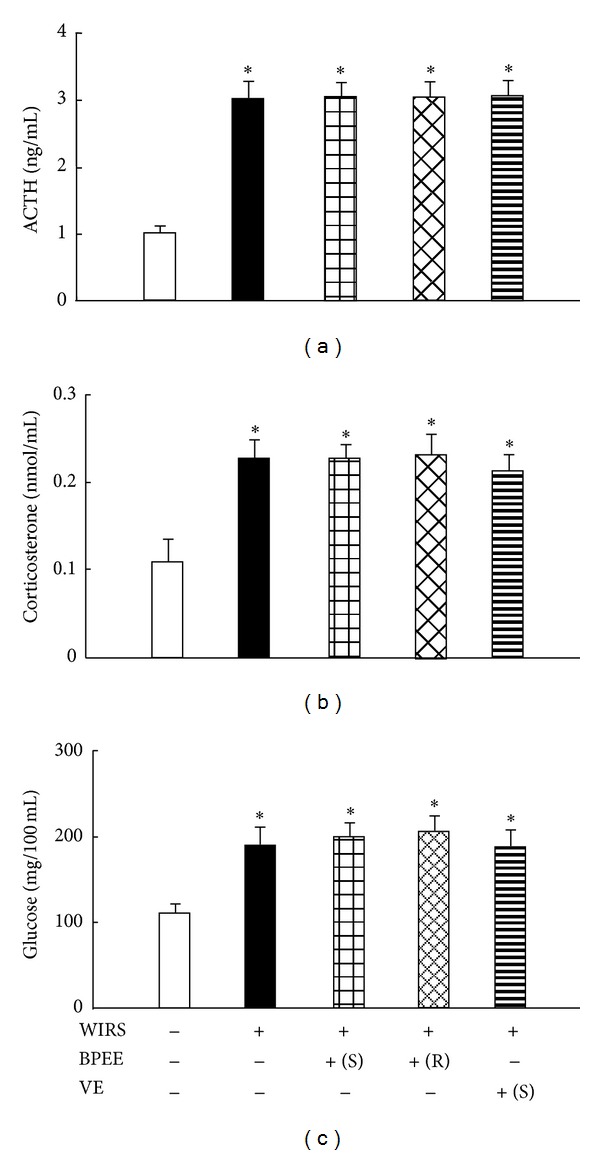
Effects of repeated or a single BPEE preadministration and a single VE preadministration repeated or a single BPEE preadministration on serum ACTH (a), CORT (b), and glucose (c) concentrations in rats with WIRS. Experimental condition and explanation are the same as described in the legend of Figure 1 except that serum ACTH, CORT, and glucose were assayed as described in Section 2. Each value is a mean ± S.D. (*n* = 8 for each group). *P < 0.05 (versus control rats without WIRS); ^#^ *P* < 0.05 (versus rats with WIRS alone).

Serum ACTH, CORT, and glucose concentrations were significantly higher in rats with 6 h of WIRS alone than those in control rats without WIRS (Figure 5). When rats preadministered with BPEE in a repeated manner and a single manner or rats preadministered with VE in a single manner were exposed to 6 h of WIRS, serum ACTH, CORT, and glucose concentrations in stressed rats preadministered with BPEE in a repeated manner and in a single manner or rats preadministered with VE in a single manner were not significantly different from those in stressed rats with any preadministration (Figure 5).

## 4. Discussion

Our previous report has shown that a single preadministration of BPEE (50 mg/kg, p.o.) to Wistar rats protects against gastric mucosal lesions induced by 6 h of WIRS more effectively than a single preadministration of the extract (10 mg/kg or 100 mg/kg, p.o.) [20]. In addition, our previous report has shown that BPEE preadministered at a dose of 100 mg/kg enhances stress sensitivity in rats with 6 h of WIRS, judging from the serum levels of stress markers such as ACTH, CORT, and glucose, resulting in the less effectiveness in protecting against WIRS-induced gastric mucosal lesions [20]. This finding has suggested that when excessive BPEE is preadministered to rats with WIRS, like the case of a single preadministration of BPEE (100 mg/kg), accumulation of unknown component(s) enhancing stress sensitivity in the preadministered BPEE in the body leads to an enhancement of stress sensitivity. Accordingly, one can assume that when BPEE (50 mg/kg/day) is orally preadministered to rats with WIRS in a repeated manner, the repeatedly preadministered BPEE exerts less protective effect against WIRS-induced gastric mucosal lesions than a single preadministration of the extract (50 mg/kg) by enhancing stress sensitivity. In the present study, however, oral preadministration of BPEE (50 mg/kg/day) to rats with 6 h of WIRS for successive seven days was found to protect against WIRS-induced gastric mucosal lesions more effectively than a single oral administration of BPEE (50 mg/kg). Increased serum ACTH, CORT, and glucose concentrations in stressed rats with repeated BPEE preadministration were not different from those in stressed rats with and without a single BPEE preadministration. These results indicate that repeated preadministration of BPEE (50 mg/kg/day, p.o.) to rats for successive seven days does not enhance stress sensitivity upon exposure to 6 h of WIRS. These results also suggest that no accumulation of unknown component(s) enhancing stress sensitivity occurs in the body of rats preadministered repeatedly with BPEE (50 mg/kg/day), resulting in no enhancement of stress sensitivity upon exposure to 6 h of WIRS. A single preadministration of VE (250 mg/kg, p.o.) to rats with 6 h of WIRS protected against gastric mucosal lesions without affecting the stress response, as reported previously [19, 35].

It has been implicated that enhanced lipid peroxidation and ROS production, depletion of NPSH, VC, and VE, excessive ^•^NO production, and inflammation associated with neutrophil infiltration contribute to gastric mucosal lesion development in rats with WIRS [9–19]. NPSH present in the gastric mucosa of rats is mainly GSH which functions as a scavenger of ROS by itself and participates in the detoxification of hydrogen peroxide and/or lipid hydroperoxides via glutathione peroxidase [36]. In the present study, rats with 6 h of WIRS showed increases in gastric mucosal LPO and NOx (a maker of ^•^NO production) concentrations and MPO and XO activities and decreases in gastric mucosal NPSH, VC, and VE concentrations. A single preadministration of BPEE (50 mg/kg, p.o.) attenuated the increased gastric mucosal LPO and NOx concentrations and XO activity and the decreased gastric mucosal NPSH, VC, and VE concentrations, but not the increased gastric mucosal MPO activity, as shown in our previous report [20]. The repeated preadministration of BPEE (50 mg/kg/day) for successive seven days attenuated not only the increased gastric mucosal LPO and NOx concentrations and XO activity and the decreased gastric mucosal NPSH, VC, VE cocentrations more effectively than the single BPEE preadministration but also the increase in gastric mucosal MPO activity, like the case of VE (200 mg/kg) preadministered in a single manner. Thus, BPEE (50 mg/kg/day) preadministered in a repeated manner was found to exert antioxidant and anti-inflammatory actions more effectively than the extract (50 mg/kg) preadministered in a single manner.

Yoshizumi et al. [37] have reported that the ethanol extract of Brazilian propolis inhibits XO activity in vitro. The same authors have shown that the contents of caffeic acid phenethyl ester (CAPE), galangin, and chrysin are in trace amount in comparison with those of artepillin C and *p*-coumaric acid in the ethanol extract of Brazilian propolis and that artepillin C and *p*-coumaric acid are very weak in inhibiting XO activity in vitro in comparison with CAPE and flavonoids such as galangin and chrysin [37]. We have reported that 40 *μ*g or more of BPEE inhibits XO activity in vitro [20]. Therefore, the inhibitory effect of BPEE preadministered in a repeated manner or in a single manner on increased gastric mucosal XO activity in rats with WIRS may be caused by unknown component(s) rather than CAPE, galangin, and chrysin in the extract. The above-described difference in the ability to attenuate increased gastric mucosal XO activity in rats with WIRS between BPEE preadministered in a repeated manner and in a single manner may be due to the difference in the accumulation of the unknown component(s) in the gastric mucosa between both preadministration manners.

The ethanol extract of Brazilian propolis and artepillin C exert antioxidant activity by inhibiting lipid peroxidation and by scavenging ROS such as superoxide radical (O_2_^−•^), hydroxyl radical, and hydrogen peroxide and free radicals [21, 38–40]. Therefore, it is suggested that repeatedly preadministered BPEE (50 mg/kg/day) could attenuate increased gastric mucosal LPO concentration in rats with 6 h of WIRS through its antioxidant action, which is possibly mainly due to artepillin C present in the extract. In addition, the accumulation of artepillin C in the gastric mucosa of rats with 6 h of WIRS could be larger in the repeated BPEE preadministration than in a single BPEE preadministration, resulting in the difference in effectiveness in protecting against WIRS-induced gastric mucosal lesions between both preadministration manners.

It has been shown that excessive ^•^NO production, increased LPO production, and NPSH depletion in the gastric mucosa of rats with WIRS are mediated by iNOS increasing in the gastric mucosa [12–14]. It has also been suggested that this excessive ^•^NO generation could be associated with an increase in neutrophils infiltrating into the gastric mucosal tissue [12, 14]. It has been reported that ^•^NO derived from ^•^NO donors infused to rats increases lipid peroxidation in the gastric mucosal tissue possibly through the formation of peroxynitrite by the reaction between ^•^NO and O_2_^−•^ [41]. It is known that the ethanol extract of Brazilian propolis and artepillin C inhibit iNOS-mediated ^•^NO production under inflammatory conditions [42–44]. It is also known that the methanol extract of Brazilian propolis scavenges both O_2_^−•^ and ^•^NO directly [45]. Furthermore, it has been shown that the ethanol extract of Brazilian propolis inhibits ROS production by activated neutrophils [46]. We have reported that 10 *μ*g or less of BPEE inhibits the production of O_2_^−•^ in activated human neutrophils without scavenging the produced O_2_^−•^ [20]. As described above, BPEE (50 mg/kg/day) preadministered in a repeated manner attenuated the increased gastric mucosal NOx concentration and MPO activity in rats with 6 h of WIRS more effectively than the extract (50 mg/kg) preadministered in a single manner. Accordingly, it is suggested that BPEE (50 mg/kg/day. p.o.) preadministered in a repeated manner could protect against WIRS-induced gastric mucosal lesions in rats more effectively than the extract (50 mg/kg, p.o.) preadministered in a single manner by inhibiting excessive ^•^NO and ROS production via infiltrated neutrophils and/or by scavenging O_2_^−•^ and ^•^NO generated via infiltrated neutrophils directly in the gastric mucosa.

de Barros et al. [7] have shown that a single preadministration of the 70% ethanol extract of Brazilian green propolis (250 or 500 mg/kg, p.o.) to pylorus-ligated rats reduces the total volume, total acidity, and pH of the gastric juice and have suggested that the ethanol extract preadministered orally at a dose of 250 or 500 mg/kg could exert a protective effect against gastric ulcer in rats with 17 h of WIRS by reducing increased acid secretion. Therefore, there seems to be a possibility that BPEE (50 mg/kg/day. p.o.) preadministered in a repeated manner rather than in a single manner exerts its protective effect against WIRS-induced gastric mucosal lesions in rats through inhibition of acid secretion. Therefore, further study is needed to examine this possibility.

In conclusion, the results obtained from the present study indicate that BPEE (50 mg/kg/day, p.o.) preadministered in a repeated manner protects against WIRS-induced gastric mucosal lesions in rats more effectively than the extract (50 mg/kg, p.o.) preadministered in a single manner possibly through its antioxidant and anti-inflammatory actions, like the case of VE (250 mg/kg, p.o.) preadministered in a single manner.
